# Wheat Biofortification for Enhancing Iron, Zinc, and Protein: The Role of Mutation Breeding

**DOI:** 10.3390/plants15152286

**Published:** 2026-07-26

**Authors:** Gulina Doktyrbay, Saule Atabayeva, Saltanat Asrandina, Sabina Shoinbekova, Aigerim Zhaxybayeva, Nurgul Amangeldi, Azamat Zhaxybayev, Malika Abdulzhanova, Xiaodong Liang

**Affiliations:** 1 Department of Biotechnology, Faculty of Biology and Biotechnology, Al-Farabi Kazakh National University, Almaty 050040, Kazakhstan; gulina.kaznu@gmail.com (G.D.); saule.atabayeva@kaznu.edu.kz (S.A.); saltanat.asrandina@kaznu.edu.kz (S.A.); sabina.shoinbekova@kaznu.edu.kz (S.S.); 2 Department of Chemistry, Institute of Natural Sciences, L.N. Gumilyov Eurasian National Univeristy, Astana 010008, Kazakhstan; zhaxybayeva_ag_1@enu.kz; 3 Department of Pre-University Education, Farabi University, Almaty 050040, Kazakhstan; nnurka87@mail.ru; 4 Department of Biology, School of Sciences and Humanities, Nazarbayev University, Astana 010000, Kazakhstan; aza.zhaxybayev@gmail.com; 5 Institute of Crop Research, Xinjiang Academy of Agricultural Sciences, Urumqi 830091, China; lxdxj@126.com

**Keywords:** gamma irradiation, micronutrient enrichment, nutritional quality, bioavailability, induced mutagenesis, genome editing, hidden hunger, agronomic traits

## Abstract

Biofortification of wheat has emerged as a sustainable strategy to combat global micronutrient deficiencies, particularly iron (Fe) and zinc (Zn) deficiency, while simultaneously improving grain protein quality. Among available approaches, mutation breeding has gained renewed attention as a non-transgenic tool capable of generating novel genetic variability for nutritional enhancement. This review is based on a comprehensive analysis of peer-reviewed literature retrieved from major scientific databases, including Web of Science, Scopus, PubMed, and Google Scholar. Studies published between 2005 and 2025 were critically evaluated to compare the effectiveness, advantages, limitations, and future prospects of wheat biofortification approaches. This review critically evaluates the role of mutation breeding in wheat biofortification and compares its effectiveness with conventional breeding, agronomic biofortification, and genome editing technologies. Evidence from published studies indicates that gamma-induced mutant lines have achieved significant increases in grain Fe and Zn concentrations, as well as improvements in storage protein composition, without regulatory constraints associated with transgenic methods. However, variability in genetic stability, potential yield penalties, and genotype × environment interactions remain important limitations. Overall, integrating mutation breeding with advanced molecular tools and agronomic practices offers a promising strategy for developing nutrient-enriched wheat varieties and enhancing global food and nutritional security.

## 1. Introduction: Global Burden of Iron, Zinc and Protein Deficiency

Micronutrient malnutrition, often described as “hidden hunger,” remains a major global public health challenge despite improvements in overall food production. Iron deficiency affects more than 1.6 billion people worldwide and continues to be the leading cause of anemia, particularly among women of reproductive age and children under five years [1,2]. Globally, anemia prevalence among women aged 15–49 years remains approximately 29–30%, with the highest burden observed in South Asia and Sub-Saharan Africa [1,3]. Iron deficiency is estimated to account for nearly half of anemia cases in low-income countries [2].

Zinc deficiency is similarly widespread, with an estimated 17–30% of the global population at risk of inadequate zinc intake [4,5]. Diets heavily reliant on cereals and legumes with low bioavailable zinc content contribute significantly to this deficiency [5]. Zinc insufficiency is strongly associated with impaired immune function, increased susceptibility to infectious diseases, and growth retardation in children [4]. According to the latest UNICEF estimates (2025), stunting remains a major global public health challenge, affecting 150 millions of children under five years of age worldwide and closely linked to chronic micronutrient deficiencies, including zinc insufficiency [6].

Protein-energy malnutrition remains a persistent concern in many low- and middle-income countries. Although global food production has increased, persistent food price inflation has severely limited access to affordable healthy diets, leaving dietary protein quality and amino acid balance suboptimal in populations heavily dependent on cheaper, cereal-based staple foods [7]. In such dietary systems, caloric sufficiency does not guarantee adequate intake of essential micronutrients or high-quality protein, creating a dual burden of energy adequacy and nutrient inadequacy [8].

The economic consequences of micronutrient deficiencies are substantial. Iron deficiency alone has been associated with productivity losses equivalent to 2–5% of gross domestic product (GDP) in some developing countries [9]. Moreover, according to the WHO Global Anaemia Estimates (2025 Edition), 30.7% of women aged 15–49 years were affected by anaemia globally. Despite ongoing efforts, only 18 countries are on track to achieve the global target of a 50% reduction in anaemia by 2030 [1]. These data emphasize that hidden hunger is not merely a nutritional issue but also a socioeconomic constraint affecting national development and public health systems.

Cereal-based dietary patterns dominate food consumption in many parts of Asia and Africa, where cereals provide over 60% of daily caloric intake [8,10]. However, intrinsic iron and zinc concentrations in staple cereals are often insufficient to meet recommended dietary allowances, and their bioavailability is further limited by antinutritional factors such as phytic acid [10]. This mismatch between caloric availability and micronutrient adequacy underscores the urgent need for sustainable, agriculture-based strategies to enhance the nutritional density of staple crops.

These global nutritional challenges highlight the urgent need for sustainable strategies that improve the nutritional quality of staple crops. Among the available approaches, plant breeding has emerged as one of the most effective long-term solutions by increasing the intrinsic concentrations of essential micronutrients without requiring major changes in dietary habits. Wheat, as one of the world’s most widely consumed staple crops, represents an ideal target for biofortification. Conventional breeding, mutation breeding, agronomic biofortification, and genome editing each contribute to enhancing grain iron, zinc, and protein content, although they differ in efficiency, cost, regulatory acceptance, and long-term sustainability. Therefore, this review focuses on critically evaluating the contribution of mutation breeding to wheat biofortification and comparing its effectiveness with other major biofortification strategies.

This review is based on a comprehensive analysis of peer-reviewed literature retrieved from major scientific databases, including Web of Science, Scopus, PubMed, and Google Scholar. The literature search focused on studies published between 2005 and 2025 using combinations of keywords such as wheat biofortification, mutation breeding, induced mutagenesis, gamma irradiation, iron, zinc, protein quality, genome editing, and agronomic biofortification. Priority was given to original research articles, review papers, and reports published in high-quality international journals.

Therefore, this review critically evaluates the role of mutation breeding in wheat biofortification, compares its effectiveness with other major biofortification approaches, and discusses its potential to enhance grain iron, zinc, and protein concentrations.

## 2. Wheat as a Strategic Vehicle for Nutritional Improvement

Given these widespread nutritional challenges, biofortification of staple crops has emerged as a promising strategy. Wheat (*Triticum aestivum* L.) is one of the most widely cultivated cereal crops globally and serves as a primary staple food for approximately 3.1–3.2 billion people [7,11]. Global wheat production reached approximately 800 million tonnes in 2024–2025, accounting for nearly 20% of total human caloric intake and about 20% of global protein consumption [11,12]. Major wheat-producing regions include the European Union, China, India, Russia, and the United States, which together account for approximately 65% of global wheat production according to recent FAO estimates [11]. Given its widespread consumption and central role in food systems, wheat represents an ideal target crop for large-scale nutritional improvement interventions.

Despite its caloric importance, the intrinsic micronutrient concentrations in modern wheat cultivars are often insufficient to meet dietary requirements in cereal-dependent populations [13]. Reported grain iron (Fe) concentrations in widely cultivated wheat varieties typically range between 25–40 mg kg^−1^, while zinc (Zn) concentrations commonly vary from 20–35 mg kg^−1^ under standard agronomic conditions [14,15]. These levels are below the target concentrations recommended by biofortification programs such as HarvestPlus [14].

Grain protein content in wheat generally ranges from 10–14%, depending on genotype, environment, and nitrogen management [16,17]. However, improvements in yield over the past decades have often been associated with a “dilution effect,” whereby increased grain yield correlates with reduced micronutrient density [13,18]. This inverse relationship poses a significant breeding challenge, as enhancing nutritional quality must not compromise yield stability or agronomic performance.

Furthermore, the nutritional contribution of wheat extends beyond total mineral concentration to include mineral bioavailability. Iron and zinc in wheat grains are frequently bound to phytic acid in the aleurone layer, limiting intestinal absorption in humans [19]. Therefore, effective nutritional improvement strategies must consider not only concentration enhancement but also mineral partitioning and reduction in antinutritional factors.

Given its global reach, established breeding infrastructure, genetic diversity, and storage stability, wheat represents an ideal vehicle for large-scale, agriculture-based nutritional interventions [14,20]. Unlike dietary supplementation programs, which require continuous financial and logistical support, genetic improvement of wheat provides a sustainable, long-term solution with potential multigenerational impact. Consequently, wheat biofortification has emerged as a central pillar in global strategies aimed at addressing hidden hunger. To achieve these nutritional targets, several complementary biofortification strategies have been developed. Table 1 summarizes the nutritional characteristics and biofortification targets of wheat grain.

Although considerable progress has been achieved in wheat biofortification, several biological and agronomic constraints continue to limit the consistent accumulation of iron, zinc, and protein in grain [13,14]. Micronutrient uptake depends not only on soil nutrient availability but also on root architecture, transporter activity, xylem and phloem transport, remobilization during grain filling, and interactions with antinutritional compounds such as phytic acid [10,19]. Furthermore, grain micronutrient concentration is strongly influenced by genotype × environment interactions, climatic conditions, soil properties, fertilizer management, and crop production practices [13,20]. These complex interactions often result in substantial variation in biofortification performance across environments, highlighting the need for integrated breeding, agronomic management, and molecular approaches to achieve stable nutritional improvement in wheat [13,20]. Recent advances in genomics, high-throughput phenotyping, and precision agriculture provide new opportunities to overcome these limitations and accelerate the development of nutrient-enriched wheat cultivars [20]. Collectively, these advances demonstrate that recent progress in genomics, precision phenotyping, and integrated breeding approaches has substantially improved the prospects for developing wheat cultivars with enhanced iron, zinc, and protein concentrations, although further efforts are still required to overcome genotype × environment interactions and physiological constraints.

## 3. Methods of Biofortification

Biofortification can be achieved through several complementary approaches aimed at increasing the concentration and bioavailability of essential nutrients in crop plants. These methods differ in their technical complexity, cost, regulatory requirements, and public acceptance. The most widely used strategies include conventional plant breeding, agronomic interventions, genetic engineering, and mutation breeding. Each approach offers distinct advantages and limitations depending on the target nutrient, crop species, and environmental conditions. In recent years, integrated breeding programs combining traditional and modern techniques have been increasingly adopted to maximize nutritional improvement. The following sections summarize the major biofortification methods and highlight their roles in enhancing iron, zinc, and protein content in staple crops.

### 3.1. Conventional Breeding

Conventional breeding remains one of the most established and widely accepted strategies for wheat biofortification because it exploits naturally occurring genetic variation without introducing foreign DNA [17,21]. Through hybridization, selection, and multi-environment evaluation, breeders combine high-yielding cultivars with donor parents possessing elevated concentrations of iron (Fe), zinc (Zn), or improved protein quality to develop nutritionally superior wheat varieties [18,22]. Recent advances in molecular markers and genomic selection have further accelerated the identification and incorporation of favorable alleles associated with micronutrient accumulation while maintaining agronomic performance [22,23].

**Figure 1:**
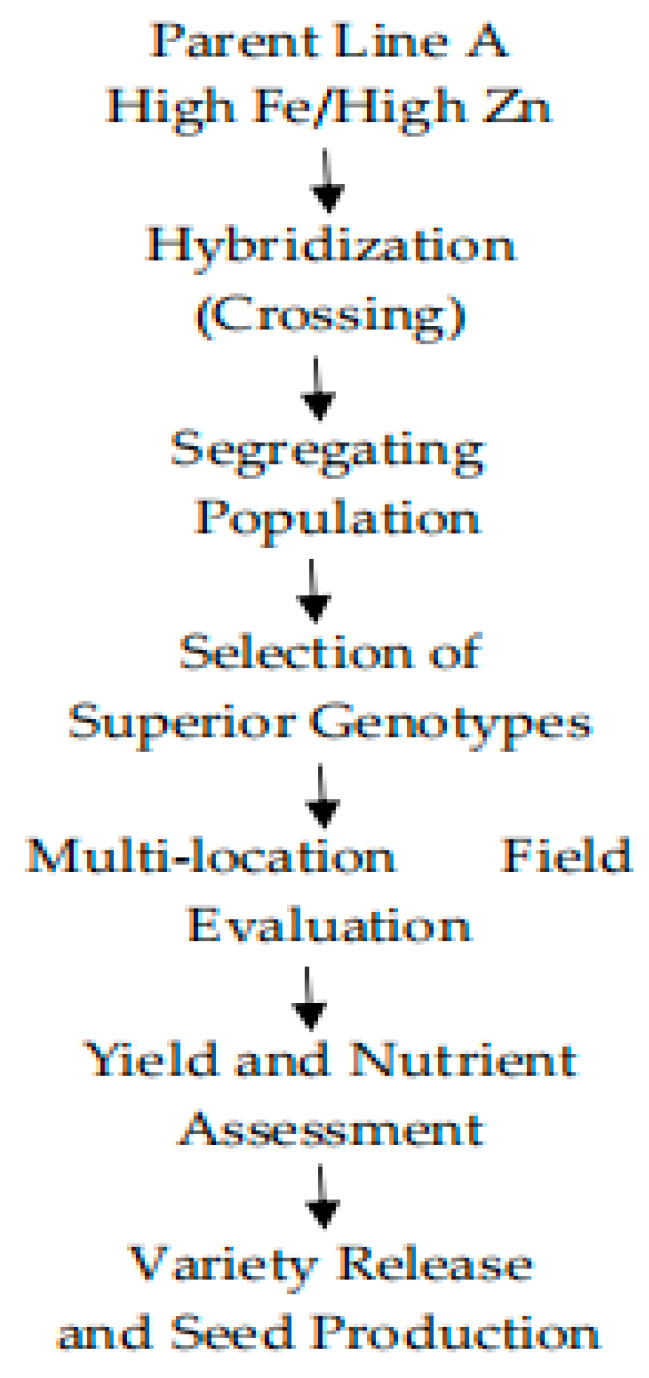
Conventional Breeding Process for Developing Biofortified Crops.

Figure 1 illustrates the conventional breeding process, beginning with parental selection and hybridization, followed by segregating population development, selection of superior genotypes, multi-location field evaluation, nutrient assessment, and eventual variety release. This stepwise approach enables breeders to simultaneously improve grain yield, adaptation, and nutritional quality under diverse environmental conditions [22,23]. The effectiveness of conventional breeding has been demonstrated in several wheat biofortification programs. HarvestPlus-supported zinc wheat varieties released in India and Pakistan increased grain Zn concentrations by approximately 8–12 mg kg^−1^ compared with conventional cultivars while maintaining competitive grain yield, providing an effective strategy to reduce zinc deficiency in populations dependent on wheat as a staple food [13,14]. In addition to zinc improvement, conventional breeding has also demonstrated considerable progress in increasing grain iron concentration. Wheat breeding programs have reported grain Fe concentrations approaching the HarvestPlus target of approximately 60 mg kg^−1^ in selected high-iron breeding lines, although achieving simultaneous improvement of iron, zinc, grain yield, and protein content remains a major breeding challenge. Furthermore, conventional breeding has successfully maintained or improved grain protein content (typically 10–14%) through the selection of superior parental lines and marker-assisted breeding, demonstrating its effectiveness in improving both nutritional quality and agronomic performance [13,14,16,17,22]. These achievements illustrate that genetic improvement through conventional breeding can substantially enhance micronutrient density without compromising agronomic performance.

Beyond wheat, successful biofortification programs further demonstrate the practical value of conventional breeding. Iron-rich bean varieties released in Rwanda and the Democratic Republic of the Congo have significantly improved dietary iron intake, while orange-fleshed sweet potato cultivars adopted in Uganda and Mozambique have markedly enhanced vitamin A status among vulnerable populations [14]. These examples confirm that conventional breeding is an effective, sustainable, and economically accessible approach for combating hidden hunger through staple crops [15].

One of the major strengths of conventional breeding is its high public acceptance, relatively low implementation cost, and compatibility with existing breeding infrastructures [22,23]. However, the development of nutrient-dense cultivars remains time-consuming because micronutrient accumulation is controlled by multiple genes and strongly influenced by genotype × environment interactions [18,23]. Nevertheless, the integration of conventional breeding with genomic tools, molecular markers, and high-throughput phenotyping has substantially improved breeding efficiency and selection accuracy [23].

Despite its widespread adoption and high public acceptance, conventional breeding remains constrained by the limited availability of natural genetic variation for grain Fe and Zn accumulation. Moreover, genotype × environment interactions frequently reduce selection efficiency, meaning that nutritionally superior genotypes identified under one environment may not consistently express the same phenotype across different growing conditions. Consequently, integrating conventional breeding with molecular markers, genomic selection, and complementary biofortification strategies is increasingly considered necessary to accelerate genetic gain while maintaining stable agronomic performance.

Overall, conventional breeding continues to represent the cornerstone of wheat biofortification. When combined with modern genomic technologies and coordinated biofortification programs, it provides a sustainable strategy for increasing iron, zinc, and protein concentrations in wheat while contributing to global food and nutrition security [13,22].

### 3.2. Genetic Engineering

Genetic engineering is an advanced biotechnological approach that enables the precise modification of plant genomes to enhance nutrient accumulation and bioavailability [14]. Unlike conventional breeding, this method allows scientists to directly introduce, modify, or regulate specific genes responsible for micronutrient synthesis, transport, and storage [15]. As a result, genetically engineered crops can achieve significantly higher levels of essential nutrients in a relatively short time [16]. Recent advances in genome editing technologies such as CRISPR/Cas9 provide opportunities for precise modification of endogenous genes without introducing foreign DNA, thereby expanding the toolbox for crop biofortification.

One of the most well-known examples of genetic biofortification is Golden Rice, which has been enriched with provitamin A through the insertion of genes involved in carotenoid biosynthesis [17]. This innovation aims to address vitamin A deficiency, particularly in developing countries where rice is a staple food [18]. Similar genetic strategies have been applied to enhance iron, zinc, and folate content in crops such as wheat, maize, and cassava [19,20]. Genetic engineering also improves nutrient bioavailability by reducing antinutritional factors, such as phytic acid, which limit mineral absorption in the human body [21]. Table 2 presents examples of genetic engineering and genome editing approaches for wheat biofortification.

Through gene silencing and metabolic pathway optimization, researchers can increase the proportion of bioaccessible micronutrients in edible plant tissues [22]. Furthermore, transgenic approaches enable the stacking of multiple beneficial traits, including enhanced nutrition, stress tolerance, and disease resistance [23].

Recent advances in genome editing technologies, particularly CRISPR/Cas systems, have further expanded the potential of genetic biofortification [24]. These tools allow precise and targeted modifications without introducing foreign DNA, making them more acceptable to regulatory agencies and consumers [25]. Genome editing has been successfully applied to improve protein quality and micronutrient content in rice and wheat varieties [26,27].

Despite its advantages, genetic engineering faces several challenges related to biosafety, ethical concerns, and public perception [28]. Regulatory approval processes are often complex and time-consuming, limiting the widespread adoption of genetically modified crops [29,30]. In addition, misinformation and social resistance continue to hinder the acceptance of biotechnology-based solutions [29].

Nevertheless, international organizations and research institutions actively promote responsible genetic engineering to combat global malnutrition [31]. Integration of genetic approaches with conventional breeding and agronomic practices has been shown to maximize the effectiveness of biofortification programs [32,33]. Long-term field trials and nutritional assessments further support the safety and efficacy of genetically engineered crops [34,35].

Overall, genetic engineering represents a powerful and promising tool for improving crop nutritional quality. When applied responsibly and supported by scientific evidence, it can significantly contribute to sustainable agriculture and global food security [36].

### 3.3. Agronomic Biofortification

Agronomic biofortification refers to the application of mineral fertilizers and foliar nutrient sprays to increase the concentration and bioavailability of essential micronutrients in wheat grain [37]. Unlike conventional breeding, this strategy provides a rapid and flexible approach for improving grain nutritional quality within a single growing season while complementing genetic improvement programs [38,39].

Among agronomic practices, foliar application of zinc (Zn) and iron (Fe) fertilizers has proven to be one of the most effective methods for wheat biofortification [40]. Numerous field experiments have demonstrated that foliar Zn application increases grain zinc concentration by approximately 20–60%, whereas combined Zn and Fe fertilization can improve grain iron concentration by 10–35%, depending on wheat genotype, fertilizer formulation, application timing, and environmental conditions [41,42,43,44]. Soil application of micronutrient fertilizers may also improve grain mineral concentration, although its efficiency is generally lower than foliar application because nutrient availability is strongly influenced by soil pH, organic matter content, and moisture conditions [45,46].

Table 3 illustrates the principal agronomic approaches used for wheat biofortification, including soil fertilization, foliar nutrient application, and integrated nutrient management. These strategies enhance micronutrient uptake, translocation, and accumulation in developing wheat grains while maintaining grain yield and overall crop performance [47].

Long-term field trials conducted in major wheat-growing regions have confirmed that integrated nutrient management, combining mineral fertilizers with organic amendments and precision fertilizer application, further improves nutrient use efficiency and increases grain Zn and Fe concentrations compared with conventional fertilization practices [48,49,50,51]. Recent studies also indicate that precision agriculture technologies, including remote sensing and site-specific nutrient management, optimize fertilizer application, reduce environmental losses, and improve the economic efficiency of wheat biofortification programs [52,53,54,55].

**Table 3:** Examples of Agronomic Biofortification Practices.

Crop	Target Nutrient	Application Method	Region	Reference
Wheat	Zinc	Foliar ZnSO_4_ spray	India	[43,44]
Wheat	Iron	Soil Fe fertilization	Pakistan	[44]
Wheat	Iron, Zinc	Combined foliar application	Kazakhstan	[48]
Wheat	Zinc	Precision fertilization	China	[52]
Wheat	Iron, Zinc	Integrated nutrient management	Europe	[55]

Values/examples are representative and may vary across wheat genotypes, growing regions, and management conditions.

Despite its advantages, agronomic biofortification has several limitations. Its effectiveness depends on environmental conditions, soil characteristics, wheat genotype, fertilizer source, application timing, and crop management practices [56,57,58]. Therefore, region-specific fertilization strategies are required to maximize micronutrient accumulation while maintaining stable grain yield under different agroecological conditions [59].

Although agronomic biofortification provides rapid improvements in grain micronutrient concentration, its effectiveness is highly dependent on soil properties, climatic conditions, fertilizer source, and crop management. In regions with low rainfall or alkaline soils, fertilizer-use efficiency may decline substantially, reducing both economic efficiency and nutritional benefits. Therefore, agronomic biofortification should be regarded primarily as a complementary strategy rather than a permanent replacement for genetic biofortification. Overall, agronomic biofortification represents a practical, cost-effective, and immediately applicable strategy for enhancing zinc and iron concentrations in wheat grain. When integrated with conventional breeding, mutation breeding, and modern biotechnological approaches, agronomic biofortification substantially contributes to sustainable wheat production, improved grain nutritional quality, and the reduction in micronutrient deficiencies in human populations [60,61,62,63,64].

### 3.4. Mutation Breeding

Mutation breeding has become one of the most effective approaches for improving wheat nutritional quality while maintaining desirable agronomic performance. Unlike conventional breeding, which relies on naturally occurring genetic variation, mutation breeding creates new genetic diversity through the application of physical or chemical mutagens, thereby expanding the pool of useful alleles available for wheat improvement [65,66,67,68,69,70]. Because induced mutations are generated without introducing foreign DNA, mutation-derived cultivars are generally accepted within conventional breeding programs and have been successfully commercialized in many countries [70,71,72].

Wheat has been one of the major crops benefiting from mutation breeding due to its global importance as a staple food and the increasing demand for biofortified cultivars capable of alleviating micronutrient deficiencies. Induced mutagenesis has generated valuable genetic variability for grain iron (Fe), zinc (Zn), protein content, disease resistance, abiotic stress tolerance, and yield-related traits [71,72,73,74]. Several successful wheat breeding programs have demonstrated that mutation breeding can simultaneously improve nutritional quality and agronomic performance, making it an important component of sustainable wheat biofortification strategies [74].

Physical mutagens, particularly gamma rays, remain the most widely used mutagenic agents in wheat improvement because they efficiently induce stable mutations throughout the genome [67,71]. Gamma irradiation produces broad phenotypic variability, allowing breeders to identify mutant plants possessing desirable nutritional and agronomic characteristics after several generations of selection. Chemical mutagens, especially ethyl methanesulfonate (EMS), mainly induce point mutations and are widely applied for functional genomics studies and precision breeding [68,69]. Following mutagenesis, mutant populations are advanced through successive generations (M_1_–M_5_), where extensive phenotypic evaluation is performed to identify superior individuals with enhanced micronutrient accumulation, improved protein concentration, and favorable agronomic performance [73,74,75,76].

**Figure 2:**
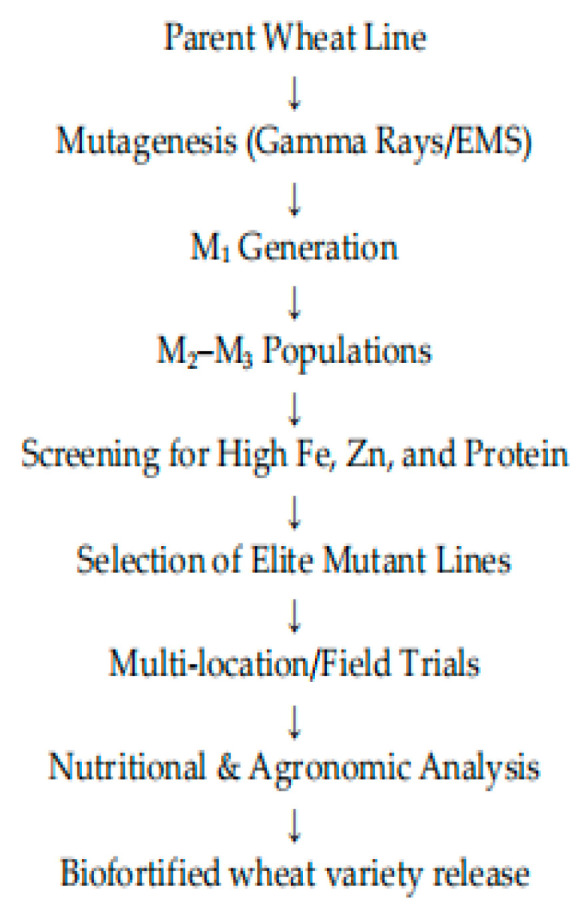
Schematic representation of the mutation breeding workflow used for the development of biofortified wheat varieties.

The general workflow of mutation breeding for wheat biofortification is illustrated in Figure 2. The process begins with the selection of elite parental wheat lines possessing desirable agronomic characteristics. Seeds are then exposed to physical mutagens such as gamma rays or to chemical mutagens such as EMS to generate large mutant populations. The first mutant generation (M_1_) is primarily used for seed multiplication, whereas subsequent generations (M_2_–M_5_) allow segregation of mutations and facilitate the identification of stable mutant lines. During these generations, extensive screening is conducted to identify plants exhibiting increased grain Fe, Zn, and protein concentrations together with desirable agronomic traits. Elite mutant lines are subsequently evaluated under multi-location field trials to confirm the stability of nutritional improvements before being released as biofortified wheat cultivars [71,72,73,74,75,76].

One of the principal objectives of mutation breeding is to improve the accumulation and bioavailability of essential micronutrients in wheat grain. Iron (Fe) and zinc (Zn) deficiencies remain among the most prevalent nutritional disorders worldwide, particularly in populations relying heavily on wheat-based diets [77,78]. Mutation breeding has proven to be an effective strategy for generating novel alleles controlling mineral uptake, translocation, remobilization, and storage, thereby increasing grain micronutrient concentrations without compromising agronomic performance [79,80].

Several studies have demonstrated that gamma irradiation can significantly enhance grain Fe and Zn concentrations in wheat mutant populations. Beneficial mutations affecting root nutrient uptake, transporter activity, and grain mineral deposition have resulted in mutant lines with superior micronutrient accumulation compared with their parental cultivars [74,78,79,80]. However, the response to gamma irradiation is strongly influenced by genotype and irradiation dose, with moderate doses generally producing a higher frequency of beneficial mutations than either very low or excessively high doses [67,74]. Consequently, optimization of irradiation conditions remains essential for maximizing breeding efficiency while minimizing undesirable mutations.

Besides increasing mineral concentration, mutation breeding also contributes to improving wheat grain protein content and overall nutritional quality. Mutations affecting nitrogen assimilation, storage protein synthesis, and grain development may simultaneously enhance protein concentration and mineral accumulation, thereby improving the nutritional value of wheat-based food products [79,80,81,82,83]. Several mutant wheat lines have demonstrated concurrent improvements in Fe, Zn, and protein content, indicating that mutation breeding can effectively target multiple nutritional traits within a single breeding program [80,82].

Another important objective of mutation breeding is the improvement of mineral bioavailability. Elevated mineral concentration alone does not necessarily translate into greater nutritional benefit because phytic acid strongly limits Fe and Zn absorption in the human digestive system. Induced mutations affecting phytic acid biosynthesis or mineral partitioning may reduce phytate accumulation while increasing mineral bioavailability, thereby enhancing the nutritional effectiveness of biofortified wheat grain [80,84]. Consequently, future mutation breeding programs should simultaneously evaluate grain Fe and Zn concentration, phytate content, and phytate-to-mineral molar ratios to achieve meaningful improvements in human nutrition.

Overall, these findings demonstrate that mutation breeding not only increases the concentration of essential micronutrients but also improves their nutritional utilization. The integration of mineral accumulation, protein enhancement, and improved bioavailability substantially increases the potential of mutation breeding as a sustainable strategy for wheat biofortification and contributes to the development of nutritionally superior wheat cultivars capable of addressing hidden hunger on a global scale [82,83,84,85].

Recent advances in molecular genetics have substantially improved the efficiency and precision of mutation breeding in wheat. The integration of mutation breeding with modern genomic tools, including Targeting Induced Local Lesions in Genomes (TILLING), molecular markers, whole-genome sequencing, and marker-assisted selection, enables the rapid identification of beneficial mutations associated with grain Fe, Zn, protein accumulation, and other agronomically important traits [75,85,86]. These technologies reduce the time required to identify elite mutant lines and facilitate the incorporation of desirable alleles into wheat breeding programs.

High-throughput phenotyping and digital image analysis have further accelerated mutant screening by allowing breeders to evaluate thousands of wheat lines for morphological, physiological, and nutritional traits with greater accuracy and efficiency [87,88]. In addition, advances in remote sensing, artificial intelligence, and machine learning provide new opportunities for predicting mutant performance under diverse environmental conditions, thereby improving selection efficiency and reducing breeding costs [73,78].

Mutation breeding has proven particularly valuable when integrated with conventional breeding strategies. Rather than replacing traditional breeding methods, induced mutagenesis expands the available genetic diversity, enabling breeders to introduce novel alleles into elite wheat cultivars through subsequent hybridization and selection [89]. This integrated approach allows the simultaneous improvement of grain nutritional quality, yield potential, disease resistance, and adaptation to environmental stresses, thereby accelerating the development of superior biofortified wheat cultivars.

Despite its considerable advantages, mutation breeding also presents several challenges. Most induced mutations are neutral or deleterious, requiring the evaluation of large mutant populations to identify individuals carrying beneficial alleles [89,90]. Furthermore, the effectiveness of mutagenesis depends on the type of mutagen, irradiation dose, wheat genotype, and environmental conditions, which may influence mutation frequency and phenotypic expression [67,71]. Consequently, extensive field evaluation across multiple environments remains essential to confirm the stability of improved nutritional traits before commercial release [90,91].

Nevertheless, the continuous development of genomic technologies, high-throughput phenotyping, and bioinformatics has substantially increased the efficiency of mutation breeding. These advances enable breeders to identify superior mutant lines more rapidly and accurately than ever before, strengthening the role of mutation breeding as a key strategy for developing nutritionally enhanced wheat cultivars capable of improving global food and nutrition security [92,93,94,95].

Future wheat biofortification programs are expected to benefit from the integration of mutation breeding with emerging genomic technologies. The combination of induced mutagenesis with genomic selection, genome-wide association studies (GWAS), transcriptomics, metabolomics, and precision phenotyping will facilitate the identification of genes regulating iron (Fe), zinc (Zn), protein accumulation, and mineral bioavailability [91,92,95]. These integrated approaches will improve breeding efficiency while reducing the time required for developing nutritionally enhanced wheat cultivars.

The application of genome editing technologies, particularly CRISPR/Cas systems, provides additional opportunities to validate beneficial mutations identified through mutation breeding and to precisely modify genes associated with micronutrient uptake, transport, storage, and grain quality [92,93,95]. Rather than replacing mutation breeding, genome editing should be considered a complementary technology that accelerates the utilization of valuable mutant alleles within wheat breeding programs.

International collaboration, germplasm exchange, and large-scale mutant population development will continue to play an essential role in expanding the genetic diversity available for wheat improvement [96,97]. At the same time, advances in artificial intelligence, machine learning, and bioinformatics will facilitate the rapid analysis of complex phenotypic and genomic datasets, enabling breeders to identify superior mutant lines with enhanced nutritional quality and agronomic performance more efficiently than conventional approaches alone [98].

Although mutation breeding has generated numerous nutritionally improved wheat lines, most induced mutations are either neutral or deleterious, requiring the screening of very large mutant populations to identify superior genotypes. Furthermore, mutation effects remain highly genotype-dependent, and the stability of improved nutritional traits must be validated across multiple environments before commercial deployment. Consequently, future research should also prioritize the simultaneous improvement of grain micronutrient concentration and bioavailability. Increasing grain Fe and Zn concentrations alone is insufficient unless these minerals are efficiently absorbed by the human body. Therefore, future mutation breeding programs should evaluate grain Fe and Zn concentrations together with protein content, phytate concentration, and phytate-to-mineral molar ratios to maximize the nutritional benefits of biofortified wheat [78,96,99].

Overall, mutation breeding remains one of the most practical and sustainable approaches for wheat biofortification because it generates novel genetic diversity without introducing foreign DNA. Numerous studies have demonstrated its ability to improve grain iron, zinc, protein concentration, and nutritional quality while maintaining desirable agronomic performance [79,80,81,82,83,84,85,86,87,88,89,90,91,92,93]. When integrated with conventional breeding, genomic technologies, precision phenotyping, and modern molecular tools, mutation breeding provides a powerful platform for developing climate-resilient, nutrient-dense wheat cultivars capable of contributing to global food and nutrition security and reducing hidden hunger [98,100,101]. Taken together, mutation breeding remains one of the most reliable and sustainable approaches for wheat biofortification. When integrated with conventional breeding, genomic selection, and modern biotechnological tools, it enables simultaneous improvement of grain iron, zinc, protein content, and mineral bioavailability while maintaining agronomic performance. Therefore, mutation breeding is expected to play a central role in the development of next-generation biofortified wheat cultivars.

### 3.5. Genome Editing (CRISPR)

Genome editing, particularly through CRISPR/Cas technology, represents one of the most advanced and precise methods for improving crop nutritional quality [93]. This technique enables targeted modification of specific DNA sequences, allowing researchers to regulate gene expression and metabolic pathways involved in nutrient synthesis, transport, and storage [102]. Unlike traditional genetic engineering, CRISPR-based editing does not necessarily introduce foreign genetic material, which increases its public acceptance and regulatory feasibility [103].

The CRISPR/Cas system functions by using guide RNA to direct the Cas enzyme to specific genomic regions, where precise cuts are made [104]. These cuts trigger natural DNA repair mechanisms, leading to gene knockouts, insertions, or replacements [105]. Through this process, genes associated with mineral uptake, vitamin biosynthesis, and protein composition can be efficiently modified [106]. As a result, crops with enhanced iron, zinc, provitamin A, and folate content can be developed within a relatively short period [107,108].

CRISPR technology has been successfully applied to major staple crops, including rice, wheat, maize, and soybean [109]. For example, editing genes related to phytic acid biosynthesis has improved mineral bioavailability in rice and wheat [110]. Similarly, modifications in carotenoid pathways have increased provitamin A content in maize and rice varieties [111]. These achievements demonstrate the high potential of genome editing for biofortification programs. Examples of CRISPR-based biofortification studies are presented in Table 4. For example, CRISPR-mediated modification of metal transporter genes in wheat has been shown to enhance grain iron and zinc accumulation, highlighting the potential of genome editing for wheat biofortification [103,112].

Despite its remarkable precision, genome editing still faces important technical and regulatory challenges. Off-target mutations, genotype-dependent transformation efficiency, intellectual property restrictions, and inconsistent regulatory policies among countries continue to limit its widespread application [24,113,114,115,116,117,118,119]. Consequently, successful deployment of CRISPR-based biofortification will require harmonized regulatory frameworks together with improved transformation protocols for elite wheat germplasm. In addition to nutritional enhancement, genome editing contributes to improved stress tolerance and yield stability, which indirectly support micronutrient accumulation. Edited crops frequently exhibit greater resistance to drought, salinity, and diseases, ensuring more stable production under changing climate conditions [97,113,114,115]. Furthermore, CRISPR facilitates the stacking of multiple beneficial traits within a single genotype [115].

Recent advances in high-throughput phenotyping and artificial intelligence have further strengthened CRISPR-based breeding programs, enabling rapid evaluation of edited lines and improving selection accuracy [98,120]. International collaboration and data-sharing platforms also play a key role in accelerating research progress and facilitating the global adoption of genome-editing technologies [121].

Overall, genome editing through CRISPR technology represents a transformative approach for crop biofortification. When supported by robust scientific evidence, transparent regulation, and public engagement, it can significantly enhance global nutrition, agricultural sustainability, and food security [122,123].

### 3.6. Comparative Economic and Practical Evaluation of Biofortification Approaches

The practical implementation of wheat biofortification strategies depends not only on their effectiveness in increasing grain micronutrient concentrations but also on their economic feasibility, regulatory requirements, scalability, and long-term sustainability. Each biofortification approach presents distinct advantages and limitations that influence its suitability for different agricultural systems and breeding objectives [121,123]. The comparative economic and practical characteristics of the major wheat biofortification approaches are summarized in Table 5.

Conventional breeding remains one of the most cost-effective and widely accepted approaches because it requires relatively low investment and faces minimal regulatory restrictions. However, the development of nutrient-enriched cultivars typically requires multiple breeding cycles, making this strategy relatively time-consuming despite its high public acceptance and broad adaptability [124,125]. Agronomic biofortification provides a rapid solution through fertilizer application and soil management practices, enabling immediate improvements in grain mineral concentration. Nevertheless, its effectiveness depends heavily on environmental conditions, fertilizer availability, repeated field applications, and production costs, which may reduce long-term economic sustainability, particularly in low-income agricultural regions [126,127].

Mutation breeding represents an intermediate strategy that combines relatively moderate development costs with permanent genetic improvement. Once desirable mutant lines have been identified and released, farmers can cultivate improved varieties without recurring investment in micronutrient fertilizers. In addition, mutation-derived varieties are generally exempt from the strict regulatory requirements associated with genetically modified crops, facilitating their commercial adoption in many countries [124]. Numerous successful wheat mutant cultivars with enhanced grain iron, zinc, and protein content demonstrate the practical value of mutation breeding for sustainable biofortification programs [96,97].

Advanced biotechnological approaches, including genome editing and genetic engineering, offer unprecedented precision for modifying genes involved in mineral uptake, transport, storage, and phytic acid metabolism. These technologies substantially reduce breeding time and enable targeted improvement of nutritional traits. However, they require sophisticated laboratory infrastructure, highly trained personnel, considerable financial investment, and, in many countries, complex regulatory approval procedures. Public acceptance of genetically engineered crops also remains variable across regions, limiting their immediate large-scale implementation despite their considerable scientific potential [122,128].

Overall, no single biofortification strategy is universally optimal. Instead, the most effective approach depends on available resources, breeding objectives, production systems, and national regulatory frameworks. In practice, integrated breeding strategies that combine conventional breeding, mutation breeding, agronomic management, and modern genomic technologies are likely to provide the greatest long-term improvement in wheat nutritional quality while maintaining economic feasibility and environmental sustainability [69,85,87].

## 4. Target Nutrients

Target nutrients in biofortification programs primarily include iron, zinc, iodine, selenium, and provitamin A, as these micronutrients play a crucial role in human health and development [129,130]. Iron is essential for hemoglobin formation and oxygen transport in the blood, while zinc supports immune function, growth, and enzyme activity [131]. Among target micronutrients, iron and zinc receive particular attention in wheat biofortification programs due to their widespread deficiencies and the naturally low bioavailable concentrations present in wheat grain. Deficiencies in these minerals are widespread in developing countries and remain a major public health concern worldwide [132].

Iodine is necessary for thyroid hormone synthesis and proper brain development, particularly during pregnancy and early childhood [133]. Selenium functions as an antioxidant and contributes to immune defense and metabolic regulation [134]. Provitamin A, which is converted into vitamin A in the human body, is vital for vision, skin health, and resistance to infectious diseases [130]. Insufficient intake of vitamin A can lead to severe health problems, including night blindness and increased mortality among children [132].

Biofortification strategies aim to increase the concentration and bioavailability of these nutrients in staple crops such as rice, wheat, maize, beans, and sweet potato [135]. Improving nutrient content alone is not sufficient; the nutrients must also be efficiently absorbed by the human body [130]. Therefore, reducing antinutritional compounds, such as phytic acid, is an important objective in crop biofortification programs [136].

Target nutrient selection is influenced by regional dietary patterns, soil properties, and climatic conditions [137]. Consequently, biofortification initiatives must be tailored to local nutritional needs and agricultural environments [131].

Overall, focusing on key micronutrients enables biofortification programs to address hidden hunger effectively and improve long-term public health outcomes [130,132]. Integrating nutritional science with plant breeding and biotechnology ensures that biofortified crops deliver sustainable and measurable benefits to vulnerable populations [132,137].

## 5. Benefits of Biofortification

Biofortification offers numerous advantages as a sustainable strategy for improving human nutrition and reducing micronutrient deficiencies worldwide [125,126]. One of its main benefits is long-term effectiveness, as biofortified crops continue to provide enhanced nutritional value across multiple growing seasons without requiring continuous external inputs [126]. This makes biofortification more cost-efficient compared to supplementation and industrial food fortification programs [127].

Another important advantage is its wide population coverage, particularly in rural and low-income communities where access to diverse diets and fortified foods is limited [125]. Since biofortified crops are incorporated into daily diets, they do not require major changes in food consumption habits, which improves acceptance and long-term impact [128]. Moreover, farmers can easily integrate biofortified varieties into existing agricultural systems, supporting local food production and income generation [126].

Mutation breeding has made a substantial contribution to the practical implementation of wheat biofortification by generating novel genetic variability for grain iron, zinc, and protein improvement. Unlike conventional breeding, induced mutagenesis enables breeders to develop nutritionally superior wheat cultivars while preserving desirable agronomic performance. Numerous mutation-derived wheat varieties have demonstrated increased micronutrient concentrations and improved nutritional quality, highlighting the important role of mutation breeding in reducing hidden hunger and supporting sustainable food security [96,125,126,130,131].

Biofortification also contributes to improved public health outcomes by reducing the prevalence of anemia, vitamin A deficiency, and weakened immune function [126]. Research indicates that regular consumption of biofortified crops significantly increases micronutrient intake among vulnerable populations, especially women and children [125,126,129]. This leads to enhanced cognitive development, physical growth, and overall productivity.

Unlike agronomic biofortification, which requires repeated fertilizer applications during each growing season, mutation breeding provides permanent genetic improvements that are stably inherited across generations. This long-term genetic stability makes mutation breeding one of the most sustainable and cost-effective strategies for wheat biofortification. In addition to increasing grain Fe, Zn, and protein concentrations, mutation breeding creates valuable genetic resources that can be readily integrated with conventional breeding, marker-assisted selection, genomic selection, and genome-editing technologies. Consequently, mutation breeding not only improves the nutritional quality of wheat but also accelerates the development of resilient, nutrient-dense cultivars capable of contributing to global food and nutritional security [91,92,93,94,95,96,97,98].

Another major advantage of mutation breeding is its compatibility with conventional breeding, marker-assisted selection, genomic selection, and genome editing. Elite mutant wheat lines enriched with iron, zinc, and protein can be incorporated into breeding programs to combine superior nutritional quality with high yield, disease resistance, and environmental adaptation. This integrated strategy accelerates the development of biofortified wheat cultivars with improved nutritional value and long-term agricultural sustainability [120,121,122,123,124].

In addition, biofortification promotes agricultural sustainability by reducing dependence on chemical supplements and imported fortified products [126]. It encourages the use of locally adapted crop varieties and supports environmentally friendly farming practices [125,127]. When combined with breeding and biotechnology approaches, mutation breeding significantly enhances the effectiveness of wheat biofortification by expanding genetic diversity and facilitating the development of nutrient-dense cultivars. Therefore, mutation breeding should be regarded as one of the key technologies supporting sustainable wheat biofortification, improving grain Fe, Zn, and protein concentrations while contributing to global food and nutrition security [124,125,126,127].

## 6. Challenges and Limitations

Despite its significant potential, biofortification faces several challenges and limitations that may affect its long-term effectiveness and large-scale adoption [124,138]. One of the major constraints is nutrient bioavailability, as increased nutrient concentration in crops does not always guarantee efficient absorption in the human body [136]. The presence of antinutritional factors, such as phytic acid and polyphenols, can reduce the bioaccessibility of minerals, limiting their health benefits.

Environmental factors also play a crucial role in determining the success of biofortification programs [129]. Soil composition, climate variability, and water availability influence nutrient uptake and accumulation in plant tissues. In regions with poor soil fertility or extreme weather conditions, biofortified crops may fail to achieve their targeted nutritional levels.

Regulatory and policy-related barriers further restrict the widespread implementation of biofortification, particularly for genetically engineered and genome-edited crops [124]. Approval procedures are often complex and time-consuming, delaying commercialization and farmer adoption. In addition, differences in regulatory frameworks among countries create challenges for international seed distribution and technology transfer.

Public perception and social acceptance remain critical issues, especially regarding genetically modified organisms [124,138]. Misinformation and limited awareness can reduce consumer trust and market demand. Moreover, insufficient extension services and farmer training programs hinder the effective dissemination of biofortified varieties [129].

Overall, addressing these challenges requires integrated approaches involving scientific research, supportive policies, and public engagement. Strengthening institutional capacity, improving communication strategies, and promoting evidence-based decision-making are essential for maximizing the impact of biofortification initiatives [124,125,126,127,128].

Although these challenges affect all biofortification strategies, their magnitude varies considerably depending on the approach employed. Agronomic biofortification is highly influenced by environmental conditions and requires repeated fertilizer applications, whereas conventional breeding and mutation breeding provide more stable long-term genetic improvements but require longer development periods. Genome editing and genetic engineering offer greater precision but remain constrained by regulatory frameworks, public acceptance, and higher implementation costs. Therefore, selecting the most appropriate biofortification strategy should consider not only biological effectiveness but also economic feasibility, environmental conditions, regulatory policies, and local agricultural practices. Future research should prioritize integrated breeding strategies that combine complementary approaches to maximize nutritional gains while ensuring long-term sustainability and farmer adoption [139,140].

## 7. Case Studies

Several successful biofortification programs worldwide demonstrate the practical impact of this approach on improving human nutrition and food security [126,128]. One of the most widely recognized examples is the development and dissemination of iron-rich common beans in Eastern and Southern Africa. These varieties have significantly reduced iron deficiency among vulnerable populations, particularly women and children, by increasing dietary iron intake through staple foods [139,140].

In South Asia, zinc-enriched wheat varieties have been introduced to address widespread zinc deficiency and improve crop productivity [140]. Field trials conducted in India, Pakistan, and Bangladesh showed that these varieties not only enhanced grain zinc content but also maintained high yield stability under diverse agroecological conditions. As a result, farmers rapidly adopted zinc-biofortified wheat due to its agronomic and nutritional benefits. Similarly, recent studies on induced wheat mutant lines developed through mutation breeding demonstrated significant improvements in grain iron and zinc concentrations, highlighting the potential of biofortification for enhancing wheat nutritional quality [141].

Another notable case is vitamin A-enriched orange-fleshed sweet potato, which has been successfully promoted in several African countries [142]. Regular consumption of this crop has been associated with improved vitamin A status in children and reduced incidence of vision-related disorders. Community-based distribution programs and nutrition education played a crucial role in increasing acceptance and long-term use.

Additionally, high-provitamin A maize varieties have been implemented in Latin America and Sub-Saharan Africa [143]. These varieties contributed to improved dietary diversity and strengthened food systems in resource-limited regions. Combined with supportive policies and farmer training, such initiatives have demonstrated the scalability of biofortification programs.

Overall, these case studies highlight that well-coordinated research, extension services, and public awareness campaigns are essential for ensuring the successful adoption and sustainability of biofortified crops [140,141,142,143].

Despite these successful examples, important limitations remain. Most reported case studies have been conducted under specific environmental and management conditions, making it difficult to directly extrapolate their outcomes across diverse wheat-growing regions. In addition, many studies primarily evaluate improvements in grain micronutrient concentration, whereas long-term assessments of micronutrient bioavailability, yield stability, farmer adoption, and economic feasibility remain limited. Future case studies should therefore employ standardized evaluation criteria and multi-location field trials to facilitate robust comparisons among conventional breeding, mutation breeding, agronomic biofortification, and genome editing approaches. Such comparative evidence will improve decision-making for selecting the most appropriate strategy under different agroecological and socioeconomic conditions [144,145,146].

## 8. Future Prospects

Future prospects of biofortification are closely linked to rapid advances in modern biotechnology, molecular breeding, and digital agriculture. Genome editing technologies, particularly CRISPR/Cas systems, are expected to play a central role in accelerating the development of nutrient-enriched crop varieties by enabling precise modification of genes responsible for micronutrient accumulation and bioavailability [144]. These tools can significantly reduce breeding time and improve selection efficiency. Particular emphasis is expected to be placed on wheat biofortification, given the crop’s global importance as a staple food and its potential to alleviate iron and zinc deficiencies.

In addition, the integration of high-throughput phenotyping, artificial intelligence, imaging technologies, and big data analysis will enhance the identification of superior genotypes with improved nutritional profiles and environmental adaptability [145]. Such technologies allow breeders to better understand genotype–environment interactions and optimize breeding strategies under diverse climatic conditions.

Despite these technological advances, several important research gaps remain. Most biofortification studies continue to evaluate grain micronutrient concentration as the primary outcome, whereas relatively few investigate micronutrient bioavailability, grain protein quality, genotype × environment interactions, or long-term field stability across diverse agroecological regions. Future research should prioritize integrating nutritional quality with agronomic performance to ensure that micronutrient-enriched wheat cultivars remain productive, stable, and widely adaptable under changing climatic conditions [147,148,149].

Future biofortification programs are also expected to focus on strengthening collaboration among researchers, policymakers, farmers, and industry stakeholders. Effective extension services, farmer training programs, and public awareness campaigns will be essential to ensure the widespread adoption of biofortified crops [146]. Furthermore, supportive regulatory frameworks and transparent communication about safety and benefits will help build public trust.

Another important priority is the development of standardized evaluation frameworks for comparing conventional breeding, mutation breeding, agronomic biofortification, and genome editing. Harmonized assessment criteria covering nutritional efficiency, economic feasibility, environmental sustainability, regulatory requirements, and farmer adoption would facilitate evidence-based decision-making and improve the practical implementation of wheat biofortification programs worldwide [150,151,152].

Overall, future progress in wheat biofortification will depend not only on technological innovation but also on multidisciplinary integration of plant breeding, nutrition, agronomy, economics, and policy research. Addressing these interconnected challenges will be essential for developing biofortified wheat varieties that are nutritionally effective, economically feasible, environmentally sustainable, and broadly accepted by farmers and consumers.

**Figure 3:**
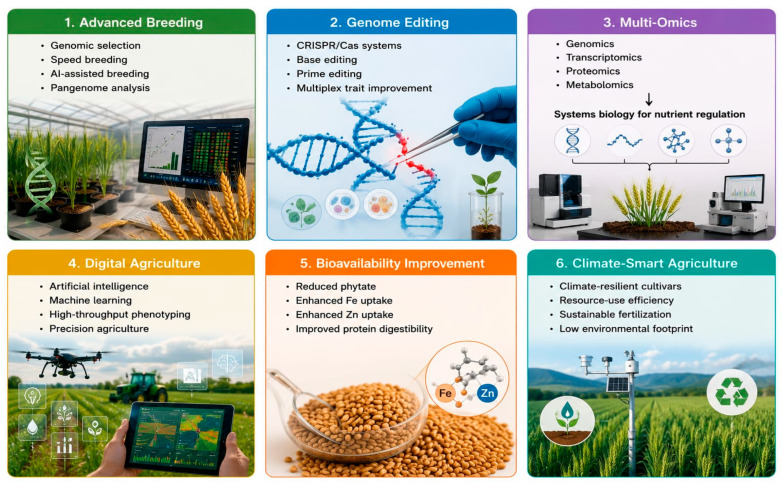
Future perspectives and integrated strategies for sustainable wheat biofortification. The schematic illustrates how advanced breeding, genome editing, multi-omics, digital agriculture, bioavailability improvement, and climate-smart agriculture collectively contribute to the development of nutrient-dense, climate-resilient, and sustainable wheat cultivars.

In our view, future progress in wheat biofortification will depend on integrating genome editing, mutation breeding, agronomic biofortification, and digital phenotyping rather than relying on a single breeding strategy. Such an integrated approach is expected to maximize nutritional gains while improving sustainability, adaptability, and long-term adoption. Figure 3 summarizes the key technological and strategic directions expected to drive future wheat biofortification programs.

## 9. Conclusions

Biofortification represents a sustainable and effective strategy for improving the nutritional quality of staple crops and addressing widespread micronutrient deficiencies. By integrating conventional breeding, mutation breeding, agronomic practices, genetic engineering, and genome-editing technologies, biofortification provides multiple pathways to enhance both the concentration and bioavailability of essential nutrients, particularly iron, zinc, selenium, and provitamin A. These approaches contribute substantially to reducing malnutrition and improving public health, particularly in developing regions.

Among the available biofortification strategies, mutation breeding has emerged as one of the most practical, efficient, and sustainable approaches for wheat improvement because it generates stable and heritable genetic variation without introducing foreign DNA. The evidence summarized in this review demonstrates that mutation breeding has successfully increased grain iron, zinc, and protein concentrations while maintaining desirable agronomic performance. Furthermore, induced mutagenesis has broadened the genetic diversity available for wheat breeding and has enabled the development of nutritionally enhanced cultivars adapted to diverse environmental conditions.

An additional advantage of mutation breeding is its strong compatibility with modern breeding technologies, including marker-assisted selection, genomic selection, high-throughput phenotyping, and genome-editing tools. This integrated approach accelerates the identification and utilization of superior alleles, thereby improving breeding efficiency and facilitating the development of climate-resilient and nutrient-dense wheat cultivars.

Future progress in wheat biofortification will depend on interdisciplinary collaboration among plant breeders, molecular geneticists, agronomists, nutritionists, and policymakers. Advances in genomics, artificial intelligence, precision phenotyping, and bioinformatics will further enhance the efficiency of mutation breeding and accelerate the release of nutritionally improved wheat varieties.

Overall, this review demonstrates that mutation breeding is not only a complementary breeding approach but also a cornerstone technology for sustainable wheat biofortification. Therefore, mutation breeding should be regarded as a key pillar of sustainable wheat biofortification and an essential component of future breeding strategies aimed at improving both nutritional quality and agronomic performance. Its proven ability to simultaneously improve grain iron, zinc, and protein concentrations while preserving desirable agronomic traits makes it an indispensable component of future breeding programs aimed at strengthening global food security, improving human nutrition, and reducing hidden hunger worldwide.

## Figures and Tables

**Table 1:** Nutritional characteristics and biofortification targets of wheat grain.

Parameter	Typical Range in Modern Cultivars	Biofortification Target	References
Iron (Fe)	25–40 mg kg^−1^	~60 mg kg^−1^	[13,14]
Zinc (Zn)	20–35 mg kg^−1^	40–50 mg kg^−1^	[14]
Protein content	10–14%	≥14% (context-specific)	[16,17]
Global production	~785–790 Mt	—	[11]
Global calorie contribution	~20%	—	[13]

Values are approximate and may vary depending on genotype and growing conditions.

**Table 2:** Examples of Genetic Engineering and Genome Editing Approaches for Wheat Biofortification.

Crop	Target Nutrient	Genetic Modification	Example Variety/Line	Reference
Wheat	Iron, Zinc	Overexpression of metal transporter genes	GM Wheat Line	[19,20]
Wheat	Iron	Ferritin gene expression	Ferritin wheat	[21]
Wheat	Zinc	CRISPR/Cas9 editing of transporter genes	Gene-edited wheat	[24]
Wheat	Protein	Genome editing of storage protein genes	Edited wheat line	[25]
Wheat	Iron, Zinc	Gene stacking	Biofortified wheat	[26]
Wheat	Iron, Zinc	Nicotianamine synthase overexpression	NAS wheat	[27]

Representative examples of wheat biofortification studies; the table is not intended to provide an exhaustive list.

**Table 4:** Applications of CRISPR/Cas Genome Editing for Wheat Biofortification.

Crop	Target Trait	Edited Gene/Pathway	Resulting Trait	Reference
Wheat	Grain protein content	Nitrogen metabolism/ storage protein genes	Increased grain protein content	[99]
Wheat	Iron bioavailability	Phytate biosynthesis genes	Improved Fe bioavailability (reduced phytic acid)	[96]
Wheat	Iron, Zinc	Metal transporter genes	Enhanced grain Fe and Zn accumulation	[103,112]
Wheat	Reduced phytic acid	Phytate biosynthesis genes	Reduced phytic acid and improved Fe/Zn bioavailability	[96]

**Table 5:** Comparative economic and practical characteristics of major wheat biofortification approaches.

Approach	Initial Cost	Time to Cultivar Release	Regulatory Complexity	Long-Term Sustainability	Main Advantages	Main Limitations
Conventional breeding	Low	Long (8–12 years)	Low	High	Low cost, high public acceptance, stable genetic improvement	Slow breeding process; limited existing genetic variation
Agronomic biofortification	Moderate	Very short (within one season)	Low	Moderate	Rapid improvement of grain mineral concentration; simple implementation	Requires repeated fertilizer application; environmental dependence
Mutation breeding	Moderate	Medium (5–8 years)	Low	High	Permanent genetic improvement; non-GM; broad public acceptance	Random mutations require extensive screening
Genome editing (CRISPR/Cas)	High	Medium (3–6 years)	High	High	Precise modification of target genes; rapid trait improvement	Expensive infrastructure; regulatory uncertainty
Genetic engineering	High	Medium–Long	Very high	High	Introduction of novel traits; multiple gene modification	Regulatory restrictions and lower public acceptance

Note: Compiled by the authors based on the cited literature.

## Data Availability

No new data were created or analyzed in this study. Data sharing is not applicable to this article.
